# In Silico Studies of Potential Selective Inhibitors of Thymidylate Kinase from *Variola virus*

**DOI:** 10.3390/ph14101027

**Published:** 2021-10-09

**Authors:** Danielle R. Garcia, Felipe R. Souza, Ana P. Guimarães, Martin Valis, Zbyšek Pavelek, Kamil Kuca, Teodorico C. Ramalho, Tanos C. C. França

**Affiliations:** 1 Laboratory of Molecular Modeling Applied to Chemical and Biological Defense, Military Institute of Engineering, Praça General Tiburcio 80, Urca, Rio de Janeiro 22290-270, Brazil; danielle.ime@gmail.com; 2 Department of Chemistry, Pontifical Catholic University of Rio de Janeiro, Rio de Janeiro 22541-041, Brazil; feliperodsouza@gmail.com; 3 Department of Chemistry, Federal University of Viçosa, Avenida P. H. Rolfs, s/n, Centro, Viçosa 36570-000, MG, Brazil; ana.paula.guimaraesime@gmail.com; 4 Department of Neurology of the Medical Faculty of Charles University and University Hospital in Hradec Kralove, Sokolska 581, 50005 Hradec Kralove, Czech Republic; martin.valis@fnhk.cz (M.V.); zbysekpavelek@email.cz (Z.P.); 5 Department of Chemistry, Faculty of Science, University of Hradec Kralove, Rokitanskeho 62, 50003 Hradec Kralove, Czech Republic; teo@dqi.ufla.br; 6 Biomedical Research Center, University Hospital in Hradec Kralove, Sokolska 581, 50005 Hradec Kralove, Czech Republic; 7 Laboratory of Computational Chemistry, Department of Chemistry, UFLA, Lavras 37200-000, MG, Brazil

**Keywords:** *Variola virus*, thymidylate kinase, smallpox, docking, molecular dynamics

## Abstract

Continuing the work developed by our research group, in the present manuscript, we performed a theoretical study of 10 new structures derived from the antivirals cidofovir and ribavirin, as inhibitor prototypes for the enzyme thymidylate kinase from *Variola virus* (*Var*TMPK). The proposed structures were subjected to docking calculations, molecular dynamics simulations, and free energy calculations, using the molecular mechanics Poisson-Boltzmann surface area (MM-PBSA) method, inside the active sites of *Var*TMPK and human TMPK (*Hss*TMPK). The docking and molecular dynamic studies pointed to structures 2, 3, 4, 6, and 9 as more selective towards *Var*TMPK. In addition, the free energy data calculated through the MM-PBSA method, corroborated these results. This suggests that these compounds are potential selective inhibitors of *Var*TMPK and, thus, can be considered as template molecules to be synthesized and experimentally evaluated against smallpox.

## 1. Introduction

Despite the declaration of the World Health Organization (WHO) that the *Variola virus* was eradicated from the world by the 1980s, smallpox is still a matter of concern. Moreover, many studies on the development of drugs against this disease have been reported in the literature [1,2,3,4,5,6,7,8,9,10,11,12,13,14,15,16]. This is due to the fact that *Variola virus* strains may have been stored in clandestine sites around the world, and their potential use for bioterrorist purposes cannot be ignored [17,18]. Furthermore, the recreation of this kind of virus has proven to be easy to carry out, considering the current technologies [19]. As viruses can survive long periods in nature, there is no guarantee that smallpox will never return as a natural pandemic [20,21]. It is also important to mention that since the 1980s the public vaccination campaigns against smallpox do not exist anymore [4,7,9], a fact that makes all of the world’s population under 40 years of age particularly vulnerable.

**Figure 1:**
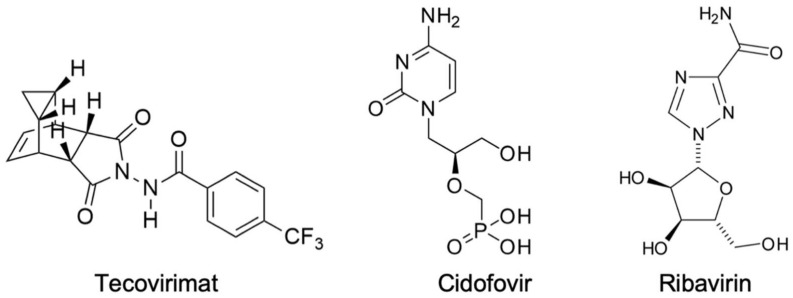
Structures of tecovirimat, cidofovir, and ribavirin.

Currently, there is only one drug approved by the Food and Drug Administration (FDA) of the United States for the treatment of smallpox. This drug, named tecovirimat (Figure 1), acts as an inhibitor of the protein complex needed for the survival of extracellular viruses, and thereby prevents its spread throughout the body from the infected cells [1,2,10]. However, despite the fact that it was approved by the FDA, tecovirimat is still in phase II of clinical trials and has not been tested in humans yet, due to the lack of patients with smallpox. It has been effective in laboratory tests to protect animals against monkeypox and rabbitpox as well as presented low toxicity to humans [1,2,10,22,23].

The risk of smallpox resurgence either, as a natural pandemic or consequence of a terrorist attack, combined with the huge number of non-immunized people in the world, and the scarcity of drugs to combat this disease, highlight the importance of the search for new drugs against it.

It is known from the literature that the Variola virus belongs to the genus Orthopoxvirus, which, similar to all poxviruses, are capable of encoding their own thymidine and thymidylate kinases (TK and TMPK) [24]. Caillat et al. [24] have detailed the sequence alignment of TMPKs from different Orthopoxviruses (vaccinia virus, smallpox, cowpox, camelpox, and monkeypox) and showed that those enzymes present highly similar amino acid sequencing, which are practically identical, except for a few residues. The majority of the different residues among the viral enzymes belong to the protein loops [24]. Therefore, the crystallographic structure of the Vaccinia virus TMPK (VaccTMPK) can be used as a template for modeling the structure of the Variola virus TMPK (VarTMPK), which is not available yet in the protein data bank (PDB) (https://www.rcsb.org/).

Moreover, the study by Caillat et al. [24] included data on practical experiments using compounds such as brivudine monophosphate to test their inhibition of VaccTMPK and human TMPK (HssTMPK). HssTMPK and VaccTMPK are only 42% similar in their amino acid sequence alignments and the dimer interface arrangements of both enzymes are different, as well as their active site geometry [24,25,26]. Due to these differences, as stated by the authors, VaccTMPK is able to accommodate more voluminous compounds, such as brivudine monophosphate, which stabilizes the enzyme and is phosphorylated. The specificity of the bond with brivudine monophosphate shows that selective antipox agents can be developed based on this finding. Therefore, these antivirals can also be investigated for their use in the treatment of TMPK-related diseases from other viruses of the Orthopoxvirus genus [24].

According to the literature, TMPK is responsible for the synthesis of thymidine 5′-triphosphate (TTP) based on the phosphorylation of thymidine 5′-monophosphate (TMP) [27,28]. TTP participates in DNA synthesis (as a building block) and its levels are controlled during the different phases of the cell cycle. Since TMPK is important for the biosynthesis of TTP, the enzyme is considered a molecular target for the development of antiviral drugs against many diseases, and the interruption of the metabolism of TTP can be used to stop the development of said illnesses. In addition to its direct effect on the metabolism of TTP, TMPK also interacts directly with the DNA synthesis process, since it plays an important role in the activation of DNA precursor nucleoside analogues. As a result, its inhibition directly affects the synthesis of genetic material, thus provoking the deactivation (death) of the virus [25,29,30].

Previously, we proposed VarTMPK as a potential target to the design of potential selective inhibitors and studied the behavior of known antivirals inside it, thus pointing to the relevant residues to be targeted in the drug design [7]. Next, we proposed the structures of 10 potential VarTMPK inhibitors based on theoretical studies of another series of antivirals [4,8,24,26]. The best ranked compounds after docking and MD simulations studies were, then, selected to design a new series of compounds based on alterations of their structural features, having in mind the synthetic viability and selectivity towards VarTMPK [9]. A few compounds of this new series presented promising theoretical results [9].

In the present work, we performed the theoretical study of 10 new compounds whose design was based on structural modifications of the antivirals cidofovir and ribavirin (Figure 1), seeking the application of these new compounds against the Variola virus. Cidofovir and ribavirin were chosen to serve as templates for this study, since they have already been extensively studied and validated for diseases caused by Orthopoxviruses. Therefore, they have much data available in specialized databanks [31,32].

Maintaining the main idea of our previous studies [4,7,9], the objective of this work was to minimize the structural complexity of the template compounds, in order to make the synthesis of these structures more feasible and less cumbersome.

To analyze the binding modes and selectivity of the proposed compounds inside *Var*TMPK and *Hss*TMPK, the molecular docking method was used [33,34]. In order to assess their dynamical behavior and corroborate the docking achievements, molecular dynamics (MD) simulations rounds were performed on the best poses obtained from docking. Finally, free energy calculations, applying the molecular mechanics Poisson-Boltzmann surface area (MM-PBSA) method, were performed on the most promising compounds to verify the effectiveness, reliability, and selectivity of their binding inside each enzyme [35,36,37,38,39,40,41,42,43,44,45].

## 2. Results

### 2.1. Docking Calculations

Table 1 lists the active site residues of *Var*TMPK and *Hss*TMPK. It is possible to see that seven of these 14 residues are different. This 50% difference highlights the possibility of designing selective inhibitors for *Var*TMPK.

Similar to our previous works [4,9], the ionization states of the compounds used in this study corresponded to the predominant microspecies at physiological conditions (pH = 7.4). According to the chemicalize server (www.chemicalize.com), our compounds except compound 1, are 100% in the neutral form at pH 7.4. The dominant microspecies for compound 1 predicted by chemicalize (www.chemicalize.com) is the one negatively charged in the phosphate group, with 74% of prevalence at pH 7.4. Moreover, according to the chemicalize server (www.chemicalize.com), all of our compounds meet the Lipinski’s criterion of drug likeness [46], as shown in Table 2.

**Figure 2:**
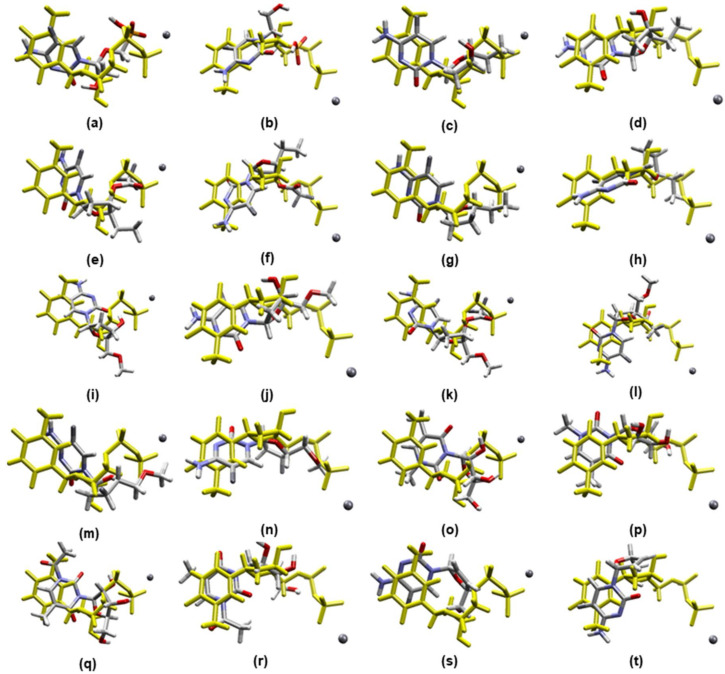
Best molecular docking poses for the prototypes inside *Var*TMPK and *Hss*TMPK: (**a**) *Var*TMPK/compound **1**, (**b**) *Hss*TMPK/compound **1**, (**c**) *Var*TMPK/compound **2**, (**d**) *Hss*TMPK/compound **2**, (**e**) *Var*TMPK/compound **3**, (**f**) *Hss*TMPK/compound **3**, (**g**) *Var*TMPK/compound **4**, (**h**) *Hss*TMPK/compound **4**, (**i**) *Var*TMPK/compound **5**, (**j**) *Hss*TMPK/compound **5**, (**k**) *Var*TMPK/compound **6**, (**l**) *Hss*TMPK/compound **6**, (**m**) *Var*TMPK/compound **7**, (**n**) *Hss*TMPK/compound **7**, (**o**) *Var*TMPK/compound **8**, (**p**) *Hss*TMPK/compound **8**, (**q**) *Var*TMPK/compound **9**, (**r**) *Hss*TMPK/compound **9**, (**s**) *Var*TMPK/compound **10**, (**t**) *Hss*TMPK/compound **10**.

**Figure 3:**
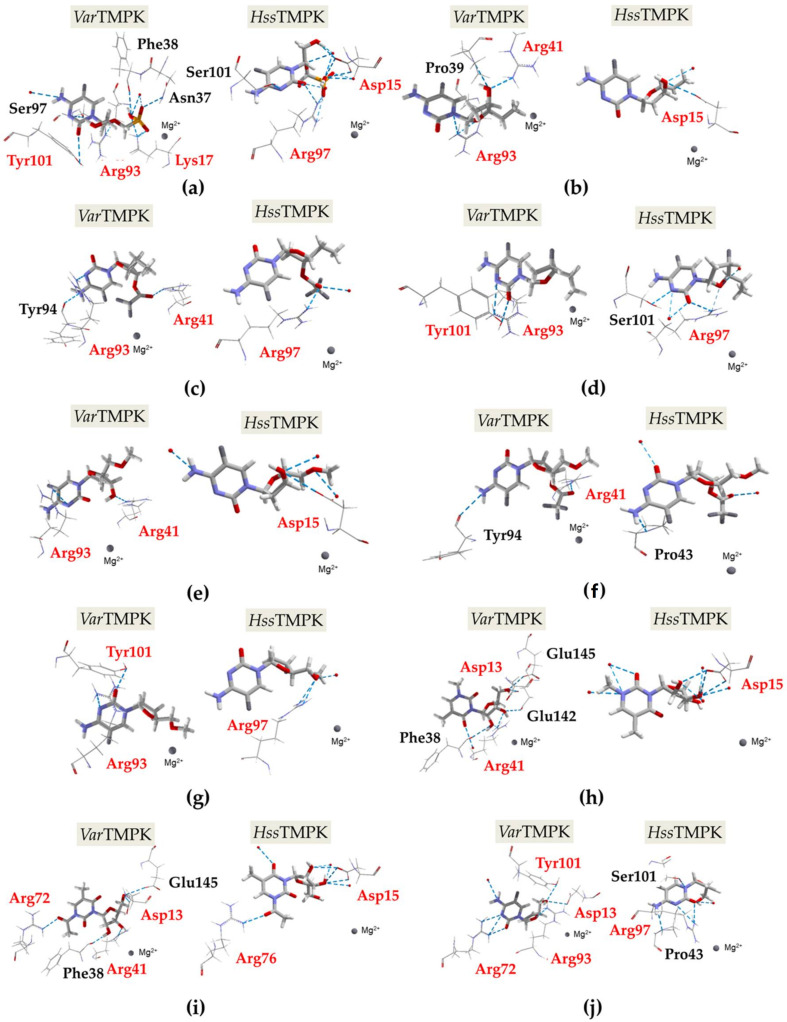
Interactions observed for the best poses obtained inside the *Var*TMPK and *Hss*TMPK active sites: (**a**) Compound **1**, (**b**) compound **2**, (**c**) compound **3**, (**d**) compound **4**, (**e**) compound **5**, (**f**) compound **6**, (**g**) compound **7**, (**h**) compound **8**, (**i**) compound **9**, (**j**) compound **10**.

The docking studies were meant to identify the compounds with better interactions and higher selectivity towards *Var*TMPK. The best poses for each compound were selected in accordance with the best (more negative) interaction energies inside *Var*TMPK and *Hss*TMPK (intermolecular and hydrogen bond) and the best superposition onto TDP. As shown in Figure 2, the poses selected in the docking studies present an optimal superposition onto TDP for both *Var*TMPK and *Hss*TMPK. These poses were analyzed according to: Their interaction energy values with the enzymes (E_interaction_) and the cofactor (E_cofactor_); the H-bond energies between the compounds and the enzymes (H_bond_); and, finally, the amino acids involved in the interactions with the compounds in both enzymes. The results obtained for those parameters are summarized in Table 3. Figure 3a–j shows the interactions observed between the proposed compounds inside *Var*TMPK and *Hss*TMPK. The active site residues of both enzymes are shown in red and the red spheres represent water molecules.

### 2.2. Molecular Dynamics Simulations

**Figure 4:**
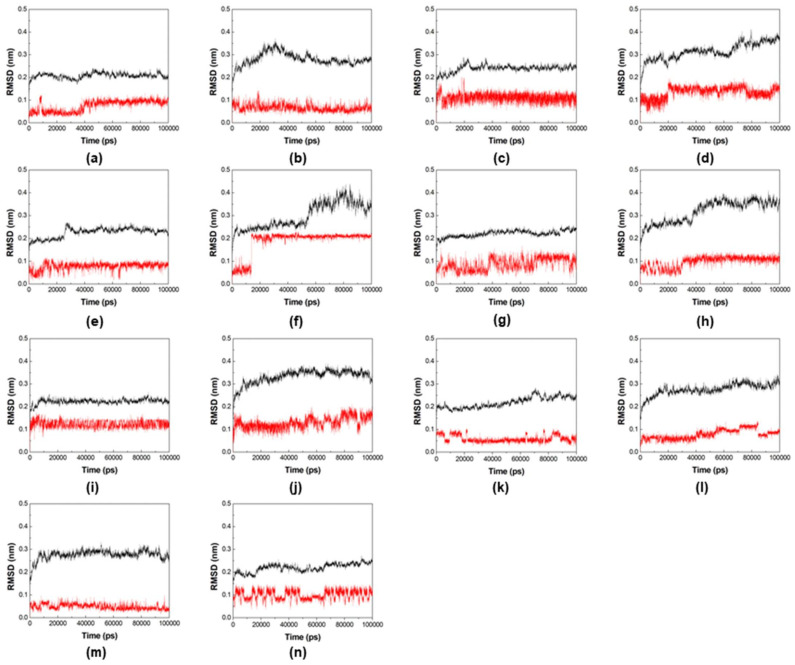
RMSD of the systems formed by the enzymes (in black) and compounds (in red): (**a**) *Var*TMPK/compound **2**, (**b**) *Hss*TMPK/compound **2**, (**c**) *Var*TMPK/compound **3**, (**d**) *Hss*TMPK/compound **3**, (**e**) *Var*TMPK/compound **4**, (**f**) *Hss*TMPK/compound **4**, (**g**) *Var*TMPK/compound **5**, (**h**) *Hss*TMPK/compound **5**, (**i**) *Var*TMPK/compound **6**, (**j**) *Hss*TMPK/compound **6**, (**k**) *Var*TMPK/compound **8**, (**l**) *Hss*TMPK/compound **8**, (**m**) *Var*TMPK/compound **9**, (**n**) *Hss*TMPK/compound **9**.

**Figure 5:**
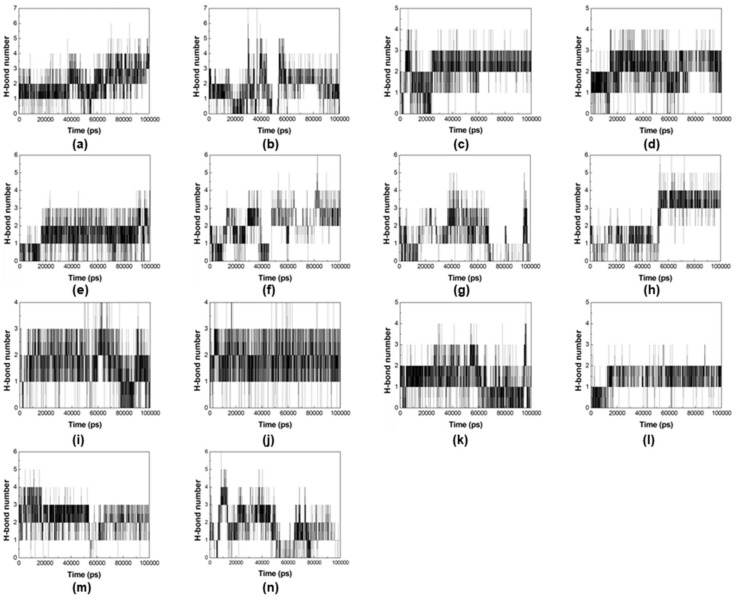
H-bonds between (**a**) VarTMPK/compound **2**, (**b**) HssTMPK/compound **2**, (**c**) VarTMPK/compound **3**, (**d**) HssTMPK/compound **3**, (**e**) VarTMPK/compound **4**, (**f**) HssTMPK/compound **4**, (**g**) VarTMPK/compound **5**, (**h**) HssTMPK/compound **5**, (**i**) VarTMPK/compound **6**, (**j**) HssTMPK/compound **6**, (**k**) VarTMPK/compound **8**, (**l**) HssTMPK/compound **8**, (**m**) VarTMPK/compound **9**, (**n**) HssTMPK/compound **9**.

The best poses of compounds **2**, **3**, **4**, **5**, **6**, **8**, and **9** were selected for additional rounds of MD simulations, since they were pointed as more selective towards *Var*TMPK by the docking studies. The results are shown in the plots of root-mean-square deviation (RMSD) and number of H-bonds formed during the MD simulations, for their complexes inside *Var*TMPK and *Hss*TMPK, shown in Figure 4 and Figure 5, respectively.

In order to validate our MD protocol, we performed three rounds of MD simulations for the complexes of compound **2** with both enzymes. The obtained plots of RMSD are shown in Figures S1–S6 of the Supplementary Material.

### 2.3. MM-PBSA Calculations

**Figure 6:**
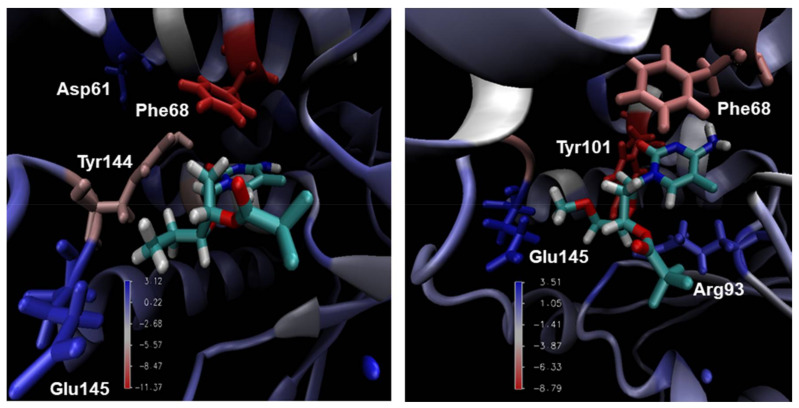
Main interactions of the complexes *Var*TMPK/compound **3** (**left**) and *Var*TMPK/compound **6** (**right**) calculated by MM-PBSA.

According to the literature, one of the limitations of docking studies lies in determining the affinities of ligand-protein complexes using calculations based solely on the poses generated for these complexes. In this regard, the MM-PBSA method tends to improve the results related to the binding energy, which is due to the fact that MM-PBSA provides more accurate results when the affinities between the ligand and protein are determined. Moreover, this is achieved by calculating the free binding energy associated with the formation of ligand-protein complexes [47] and decomposing it in the contributing components, as proposed before by Bren et al. [44,45]. Table 4 shows the average binding energy values calculated for compounds **2**, **3**, **4**, **6**, and **9** complexed with *Var*TMPK and *Hss*TMPK, while Figure 6 illustrates the favorable and unfavorable energy contributions of compounds **3** and **6** inside *Var*TMPK.

## 3. Discussion

As can be seen, Table 2 shows the toxicity and mouse carcinogenicity values of the investigated structures and the antivirals used as precursors. In short, with respect to the proposed compounds, it is important to point out that the acute toxicity values in algae calculated for all the structures were lower than the value found for cidofovir (1.19), which is not toxic. Furthermore, except for compounds **1** and **10**, all the structures had lower toxicity values in comparison with those of ribavirin (0.54).

According to the literature, the cavity of *Hss*TMPK acquired through the software Molegro virtual docker (MVD)^®^, presents a volume of 90.112 Å^3^, which is greater than the value of 76.288 Å^3^ observed for the cavity of *Var*TMPK. It is important to point out that the *Hss*TMPK cavity is more outspread and narrower. Therefore, it is expected to have issues in the entrance and/or permanence of larger inhibitors in the binding site [7].

The results in Table 3 show that all the compounds are capable of binding to the active sites of both enzymes. This is reflected by the negative values of E_interaction_ observed for all of them. Comparing the docking results obtained, we can state that compounds **2**, **3**, **4**, **5**, **6**, **8**, and **9** form more stable complexes with VarTMPK. This enables us to infer that they might show higher affinity for the active site of this enzyme, since the systems formed among these compounds and the viral enzyme presented lower values of E_interaction_, E_cofactor_, and H-bond energy. Regarding the ΔE_interaction_ values, determined based on the difference between E_interaction_ inside *Var*TMPK (lower value) and *Hss*TMPK, the compounds listed above ranked: **2** (−62.85 kcal.mol**^−^**^1^) < **5** (−55.40 kcal.mol**^−^**^1^) < **9** (−45.90 kcal.mol**^−^**^1^) < **4** (−38.57 kcal.mol**^−^**^1^) < **3** (−35.41 kcal.mol**^−^**^1^) < **6** (−27.85 kcal.mol**^−^**^1^) < **8** (−11.32 kcal.mol**^−^**^1^).

The best pose of compound 1 interacts with three active site residues of *Var*TMPK and two active site residues of *Hss*TMPK, as shown in Figure 3a. Among these residues, Arg93 from *Var*TMPK and Arg97 from *Hss*TMPK are equivalent between both enzymes.

Regarding compound **2** (Figure 3b), we observed interactions with three residues from *Var*TMPK, of which two belong to the active site, while for *Hss*TMPK, an interaction with only one residue was observed, which also belongs to the active site.

Compounds **3** (Figure 3c) and **4** (Figure 3d) interact with the same number of residues inside both enzymes. However, for *Var*TMPK, two interacting residues belong to the active site versus only one for *Hss*TMPK. Residues Arg93 from *Var*TMPK and Arg97 from *Hss*TMPK are the only equivalent interacting residues observed.

Figure 3e shows that compound 5 interacts with two active site residues from *Var*TMPK and one from *Hss*TMPK. No matching interacting residues were observed.

According to Figure 3f, compound 6 interacts solely with one active site residue from *Var*TMPK and has no interaction with the *Hss*TMPK active site residues. On the other hand, for compound **7**, Figure 3g shows interactions with two active site residues from *Var*TMPK and one from *Hss*TMPK. Arg93 from *Var*TMPK and Arg97 from *Hss*TMPK were the only equivalent interacting residues observed.

Regarding the complexes formed with compound **8** (shown in Figure 3h), we can see interactions with two active site residues from *Var*TMPK and one from *Hss*TMPK. In this case, Asp13 from *Var*TMPK and Asp15 from *Hss*TMPK are the equivalent interacting residues observed.

Figure 3i shows that compound **9** interacts with three active site residues from *Var*TMPK, and two from *Hss*TMPK. The equivalent interacting residues observed are: Asp13 (*Var*TMPK) and Asp15 (*Hss*TMPK), and Arg72 (*Var*TMPK) and Arg76 (*Hss*TMPK).

Finally, Figure 3j shows that compound **10** interacts with four active site residues from *Var*TMPK, and only one from *Hss*TMPK. The equivalent residues observed are Arg93 from *Var*TMPK and Arg97 from *Hss*TMPK.

The RMSD plots for the multiple MD simulations, performed for the systems enzyme/compound 2 (Figures S1–S6), showed the same behavior for all dynamics, with no variation above 0.1 nm. This validates our MD simulations protocol for the systems under study.

Comparing the RMSD plots in Figure 4 for both enzymes, it is possible to see that for all the systems, the RMSD values never passed 0.4 nm for the enzyme (black lines) and 0.2 nm for the ligands (red lines) during the simulated time. However, for most of the systems, both the enzyme and compounds presented more fluctuations inside *Hss*TMPK. Inside *Var*TMPK, this fluctuation never passed 0.05 nm for compounds **2**, **3**, **4**, **6**, and **9**.

Compounds **5** and **8** were the only ones showing a similar or more instable behavior inside *Var*TMPK compared to *Hss*TMPK (see Figure 4g,h,k,l). This does not corroborate with the docking results and suggests that these compounds are not selective towards *Var*TMPK.

In addition, we analyzed the number of H-bonds formed during the MD simulations for the complexes inside *Var*TMPK and *Hss*TMPK (Figure 5). The H-bonds profiles of the complexes with compounds **2** (Figure 5a,b), **3** (Figure 5c,d), **4** (Figure 5e,f), **6** (Figure 5i,j), and **9** (Figure 5m,n) show that these compounds were capable of keeping at least three H-bonds with *Var*TMPK during the whole simulated time, presenting a profile better or similar to *Hss*TMPK. Conversely, the H-bond graphs of the complexes with compounds **5** (Figure 5g,h) and 8 (Figure 5k,l) show a more unstable behavior with the prevalence of no more than two H-bonds during the whole simulation, presenting a worse (compound **5**) or similar (compound **8**) profile compared to *Hss*TMPK.

The results regarding the formation of H-bonds can be correlated to the RMSD observed during the MD simulations, and point to compounds **2**, **3**, **4**, **6**, and **9** as more selective towards *Var*TMPK. For this reason, these compounds were selected for the determination of their binding energies inside *Var*TMPK and *Hss*TMPK based on the MM-PBSA calculations.

Regarding the MM-PBSA calculations, the results in Table 4 show that for all compounds, the mean binding energy values of the complexes formed with *Var*TMPK were lower than the values determined for the complexes formed with *Hss*TMPK. These data confirm the results obtained by the docking and MD simulations, which suggested the selectivity of these compounds towards *Var*TMPK.

Moreover, the results in Table 4 show that compounds **3** and **6** stand out among the others in terms of binding energy with *Var*TMPK, which are the most stable ones. Furthermore, the difference related to *Hss*TMPK was higher than 90 KJ/mol in both cases, suggesting a high selectivity towards *Var*TMPK.

A possible explanation for the high free energy values of the complexes between *Var*TMPK and compounds **3** and **6**, is the presence of the trifluoroacetate group (–COOCF_3_) attached to the tetrahydrofuran ring. The presence of three fluorine atoms—a chemical element with high electronegativity and with isolated and free electron pairs on its structure—likely favors the selective interactions inside *Var*TMPK. Therefore, these interactions can help in maintaining the complexes formed between *Var*TMPK and compounds **3** and **6**—thus contributing to the higher stability of these systems.

## 4. Materials and Methods

### 4.1. Compounds Studied

**Figure 7:**
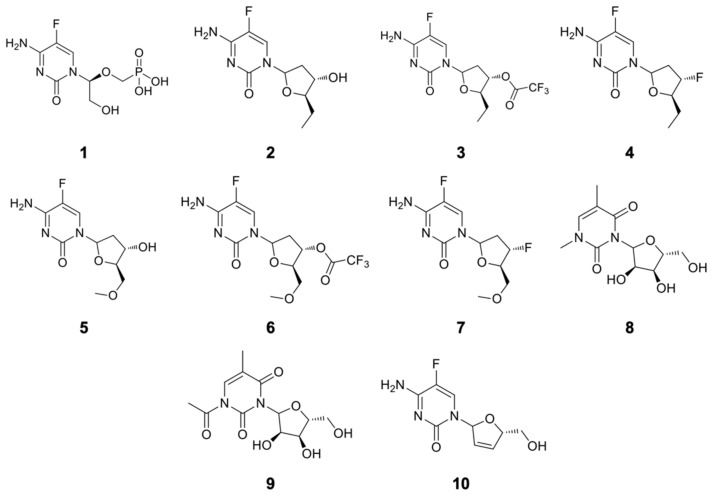
Structures of the studied compounds.

The 2D structures of the compounds studied in this work are shown in Figure 7. As mentioned above, they are all derivatives from the antivirals cidofovir and ribavirin, designed to better explore the potential selective interactions with residues of the active site of *Var*TMPK.

The ionization states of each compound in Figure 7 at physiological pH (7.4)—as well as the drug likeness criteria established according to the Lipinski’s rule of five [48]—were calculated with the aid of the Chemicalize databank (https://chemicalize.com) [46]. Their toxicity and carcinogenicity in mice were calculated with the online program PreADMET (https://preadmet.bmdrc.kr/adme-prediction/).

The tridimensional structures of these compounds, and the calculation of their atomic charges, were done through the Recife model 1 (RM1) semiempirical model [49,50], using the Spartan 08^®^ Suite software.

### 4.2. Applied Protocols

In previous works [4,7,9], the tridimensional structures of *Var*TMPK and *Hss*TMPK that were used for our studies include, respectively, the homology model complexed with TDP and the Mg^2+^ cofactor, constructed by Guimarães et al. [7], and the crystallographic structure of *Hss*TMPK complexed with TDP and Mg^2+^, available in the protein data bank (PDB) under the code 1E2G [51]. According to Guimarães et al. [7], the Ramachandran plot of the homology model showed that 99.5% of the residues were situated in the most favored regions.

The docking protocol used, as well as its validation by re-docking studies, was the same as employed by Guimarães et al. [7]. The MolDock algorithm [52] from the Molegro virtual docker (MVD^®^) was used to perform the energy calculations of the ligands inside VarTMPK and *Hss*TMPK. The binding site was restricted to spheres with radii of 6 and 10 Å, respectively, around TDP, and all the residues inside these spheres were set to be flexible. The coordinates were centered on x = 8.95, y = 22.41, and z = 0.69 for VarTMPK, and x = 13.92, y = 75.19, and z = 25.05 for *Hss*TMPK [7]. The best poses for each ligand in the viral and human enzymes were selected for further MD simulations.

The protocol comprising the application of MD simulations rounds to the best poses selected from the docking studies was based on our most recent work [9]. Each ligand was parameterized in order to be recognized by the OPLS-AA force field [53]. The enzyme/ligand complexes were put inside cubic boxes of 450 nm^3^ containing around 13,000 Tip4P type water molecules under periodic boundary conditions (PBCs), using the GROMACS 5.1.4 software [54]. The energy minimization steps used were: (1) Steepest descent with position restraint (PR) of the ligand; (2) steepest descent without PR; (3) conjugate gradients; and, lastly, (4) quasi Newton Broyden–Fletcher–Goldfarb–Shanno (L-BFGS) algorithm [55], with a minimal energy of 1 kcal.mol^−1^. Thereafter, the systems were submitted to temperature (NVT) and pressure (NPT) balancing phases in order to attain equilibrium. The equilibrated systems were, then, submitted to 500 ps of MD at 310 K with a PR for the entire system, except for water molecules, in order to ensure the equilibrium of solvent molecules, and finally, 100,000 ps of free MD simulations at 310 K without PR, with 2 fs of integration time, and a cutting radius of 10 Å for long-distance interactions [9]. All the Glu and Asp residues were assigned with negative charges, and the Lys and Arg residues were assigned with positive charges. As mentioned above, the visual molecular dynamics (VMD) software [56] was used to analyze the MD results of the systems. In order to validate our MD simulations protocol, three MD simulations were performed for the systems VarTMPK/compound 2 and *Hss*TMPK/compound 2.

The MM-PBSA method was employed to predict the free binding energies of the ligands inside the VarTMPK and *Hss*TMPK. This study allows us to infer whether the binding process is spontaneous (freeing energy) or nonspontaneous (requiring energy), thus enabling us to point to the prototype that is more interesting and promising regarding the selective inhibition of VarTMPK. The determination of the free energy of formation of the complexes, in association with the MD simulations, take into consideration three energetic terms in the calculation of binding energy: (1) Changes in the potential energy of the system in a vacuum; (2) polar and apolar solvation of the different species; and (3) the entropy related to the formation of the complexes during the gaseous phase [9,36,37,44,45]. For all the enzyme/ligand complexes, MM-PBSA calculations were performed using the g_mmpbsa tool [37] from the GROMACS package. In order to consider non-correlated frames, the structures for the free energy calculations were obtained at 500 ps each after the stabilization of the systems [9].

The parameters used for the docking, MD simulations, and MM-PBSA calculations performed in this work are summarized in Table 5.

## 5. Conclusions

According to the docking studies, it was found that only compounds **1**, **7**, and **10** did not demonstrate selectivity towards *Var*TMPK. The MD simulations studies corroborated the docking results for compounds **2**, **3**, **4**, **6**, and **9**, implying that the complexes formed between each of them and *Var*TMPK are more stable when compared to the same complexes with *Hss*TMPK. This was also observed in the further MM-PBSA calculations of the binding energies that showed lower binding values for these compounds inside *Var*TMPK than inside *Hss*TMPK. Moreover, the MM-PBSA studies pointed to compounds **3** and **6** as the most promising selective inhibitors of *Var*TMPK with the higher difference in binding energies. This is probably due to the presence of the—COOCF_3_ group on these compounds that could be favoring their stabilization inside *Var*TMPK.

In conclusion, our results suggest that five of the 10 proposed compounds, derived from the antivirals cidofovir and ribavirin, can be excellent alternatives for the development of new drugs against smallpox. Since TMPK is highly conserved amongst other Orthopoxviruses, similar to the vaccinia and monkeypox viruses, the same theoretical approach used here can be done with the TMPKs of those viruses as a proof of concept. Furthermore, the experimental evaluation of these compounds should be first performed against those viruses as a primarily model to check their potential in vitro. This would help in confirming the activity of those compounds before moving to the more complicated evaluation against the Variola virus, which will demand both an authorized laboratory and a stricter safety protocol.

## Figures and Tables

**Table 1:** Active site residues of VarTMPK and HssTMPK. The non-matching amino acids are shown in red.

VarTMPK	Asp13, Lys14, Ser15, Lys17, Thr18, Arg41, Leu53, Asn65, Phe68, Cys69, Arg72, Arg93, Tyr101, Ala102
HssTMPK	Asp15, Arg16, Ala17, Lys19, Ser20, Arg45, Leu57, His69, Phe72, Ser73, Arg76, Arg97, Phe105, Thr106

**Table 2:** Calculated values of acute toxicity, carcinogenicity (in mouse), and Lipinski’s rule of the compounds and antivirals.

Structure	Acute Toxicity (Algae)	Carcinogenicity (Mouse)	Meets Lipinski’s Rule?
1	1.10	negative	Yes
2	0.30	positive	Yes
3	0.15	positive	Yes
4	0.22	positive	Yes
5	0.51	positive	Yes
6	0.25	positive	Yes
7	0.38	positive	Yes
8	0.31	positive	Yes
9	0.37	positive	Yes
10	0.60	positive	Yes
Cidofovir	1.19	Positive	Yes
Ribavirin	0.54	negative	Yes

**Table 3:** Docking results for the compounds studied and TDP inside *Var*TMPK and *Hss*TMPK active sites.

#	E_interaction_ (kcal.mol^−1^)		E_cofactor_ (kcal.mol^−1^)		H-Bond Energy (kcal.mol^−1^)		H-Bond Interactions		∆E_int._ (kcal.mol^−1^)
	*Var*TMPK	*Hss*TMPK	*Var*TMPK	*Hss*TMPK	*Var*TMPK	*Hss*TMPK	*Var*TMPK	*Hss*TMPK	
**1**	−100.00	−128.35	−25.04	−4.13	−14.74	−15.18	Asn37 (2.91Å) Lys17 (2.08Å; 2.98Å) Phe38 (3.23Å) Arg93 (2.87Å) Tyr101 (3.06Å) Ser97 (3.36Å) H_2_O (2.67Å; 3.20Å)	Asp15 (3.10Å; 2.97Å; 2.97Å) Arg97 (2.60Å; 2.60Å; 3.05Å) Ser101 (2.93Å) H_2_O (3.21Å; 2.49Å; 2.52Å; 2.96Å; 2.94Å; 2.87Å; 3.33Å)	+28.35
**2**	−95.43	−32.58	−0.24	−0.19	−7.93	−2.50	Pro39 (3.06Å) Arg93 (2.88Å; 3.10Å) Arg41 (2.76Å)	Asp15 (3.10Å)H_2_O (3.38Å)	−62.85
**3**	−110.95	−75.54	−2.45	−0.34	−6.44	−1.58	Tyr94 (3.11Å) Arg93 (3.10Å) Arg41 (2.61Å)	Arg97 (2.65Å) H_2_O (2.84Å)	−35.41
**4**	−89.18	−50.61	−0.61	−0.53	−4.84	−2.73	Arg93 (3.10Å) Tyr101 (3.13Å)	Arg97 (2.6Å; 2.85Å) Ser101 (2.49Å) H_2_O (2.46Å; 3.43Å; 3.38Å)	−38.57
**5**	−90.77	−35.37	−1.47	−0.21	−3.86	−2.58	Arg41 (3.09Å) Arg93 (3.10Å)	Asp15 (3.10Å) H_2_O (2.45Å; 3.02Å; 3.09Å)	−55.40
**6**	−112.24	−84.39	−2.64	−0.17	−3.32	−2.50	Tyr94 (3.32Å) Arg41(2.69Å)	Pro43 (2.64Å) H_2_O (2.93Å; 3.33Å)	−27.85
**7**	−41.11	−58.87	−1.33	−0.47	−4.22	−3.70	Arg93 (3.11Å) Tyr101 (2.63Å)	Arg97 (3.10Å; 3.12Å) H_2_O (3.27Å)	+17.76
**8**	−106.20	−94.88	−0.63	−0.28	−12.93	−7.79	Phe38 (3.17Å) Arg41 (3.06Å)Glu142 (3.10Å) Asp13 (3.10Å)Glu145 (2.65Å) H_2_O (3.05Å)	Asp15 (2.72Å; 3.10Å; 3.10Å) H_2_O (3.28Å; 2.77Å; 3.21Å; 2.94Å; 3.33Å; 3.25Å)	−11.32
**9**	−120.94	−75.04	−1.23	−0.08	−12.33	−10.00	Arg72 (2.70Å) Phe38 (2.90Å) Arg41 (2.98Å) Asp13 (3.10Å) Glu145 (3.10Å)	Arg76 (2.60Å) Asp15 (3.10Å; 3.00Å; 3.00Å) H_2_O (2.67Å; 3.30Å; 2.73Å; 3.04Å)	−45.90
**10**	−44.85	−101.15	−0.80	−0.64	−8.63	−7.40	Arg72 (2.75Å; 2.88Å; 3.19Å) Arg93 (3.10Å) Tyr101 (3.10Å)Asp13 (3.10Å) H_2_O (2.84Å)	Pro43 (3.28Å) Arg97 (3.24Å; 2.67Å) Ser101 (3.24Å) H_2_O (3.36Å)	56.30
**TDP**	228.18	−195.60	−50.38	−18.13	−3.82	−13.97	Asp13, Lys17, Asn37, Arg41, Arg72, Arg93	Asp15, Arg45, Arg76, Arg97, H_2_O	

**Table 4:** MM-PBSA results for compounds **2**, **3**, **4**, **6**, and **9**.

	Binding Energies kJ.mol^−1^	
Compound	*Var*TMPK	*Hss*TMPK
**2**	−133.69 ± 6.92	−36.60 ± 3.71
**3**	−189.90 ± 3.05	−90.79 ± 3.56
**4**	−59.50 ± 4.37	−33.50 ± 4.41
**6**	−206.04 ± 12.98	−100.85 ± 2.24
**9**	−81.72 ± 2.58	−43.16 ± 4.36

**Table 5:** Parameters used in the docking and MD studies.

Docking	Software Used in Energy Calculations	Algorithm Used in Energy Calculations	Radius of the Anchoring Area	Enzyme Site Coordinates		No. of Repetitions	No. of Poses
				***Hss*** **TMPK**	***Var*** **TMPK**		
	Molegro virtual docker (MVD^®^)	MolDock [52]	6–10 Å	x = 8.95 y = 22.41 z = 0.69	x = 13.92 y = 75.19 z = 25.05	10	30
**Molecular Dynamics**	**Force Field**	**Software Used in** **Coordinate Parameters and Topologies**	**Software Used in** Construction of Cubic Boxes *	**Simulation Stage Parameters**		**Software Used to Analyze MD Results**	
				**1st**	**2nd**		
	OPLS-AA [53]	AnteChamber PYthon Parser InterfacE (AcPype) [57]	GROMACS 5.1.4 [54]	t = 500 ps T = 310 K w/ position restriction	t = 100.000 ps T = 310 K	Visual molecular dynamics (VMD) [56]	

* Parameters of cubic boxes: 450 nm^3^/1300 H_2_O/Tip4P molecules.

## Data Availability

Data is contained within the article and supplementary material.

## Supplementary Materials

The following are available online at https://www.mdpi.com/article/10.3390/ph14101027/s1, Figure S1: RMSD of the first MD simulation for the system HssTMPK/Compound 2, Figure S2: RMSD of the second MD simulation for the system HssTMPK/Compound 2, Figure S3: RMSD of the third MD simulation for the system HssTMPK/Compound 2, Figure S4: RMSD of the first MD simulation for the system VarTMPK/Compound 2, Figure S5: RMSD of the second MD simulation for the system VarTMPK/Compound 2, Figure S6: RMSD of the third MD simulation for the system VarTMPK/Compound 2.
