# Supplementary material for: In Silico Studies of Potential Selective Inhibitors of Thymidylate Kinase from Variola virus
Source: Pharmaceuticals (Basel). 2021 Oct 9;14(10):1027. doi: 10.3390/ph14101027 (PMC8537287; doi:10.3390/ph14101027)
Supplement: Supplementary file 1 [file pharmaceuticals-14-01027-s001.zip › pharmaceuticals-1307585-supplementary.pdf]

pharmaceuticals

Article

In Silico studies of potential selective inhibitors of thymidylate kinase from *Variola virus*

Danielle R. Garcia[1], Felipe R. de Souza[2], Ana P. Guimarães[3], Martin Valis[4], Zbyšek Pavelek[4], Kamil Kuca[5,6*], Teodorico C. Ramalho[5,7] and Tanos C. C. França[1,5,*]

[1] Laboratory of Molecular Modeling Applied to Chemical and Biological Defense, Military Institute of Engineering, Praça General Tiburcio 80, Urca, 22290-270, Rio de Janeiro/RJ – Brazil. danielle.ime@gmail.com (D.R.G);
[2] Department of Chemistry, Pontifical Catholic University of Rio de Janeiro, Rio de Janeiro/RJ –Brazil. feliperodsouza@gmail.com (F.R.S);
[3] Department of Chemistry, Federal University of Viçosa, Avenida P. H. Rolfs, no number, Centro, 36570-000, Viçosa/MG – Brazil; ana.paula.guimaraesime@gmail.com (A.P.G);
[4] Department of Neurology of the Medical Faculty of Charles University and University Hospital in Hradec Kralove, Sokolska 581, 50005 Hradec Kralove, Czech Republic; martin.valis@fnhk.cz (M.V); zbysekpavelek@email.cz (Z.P)
[5] Department of Chemistry, Faculty of Science, University of Hradec Kralove, Rokitanskeho 62, 50003, Hradec Kralove, Czech Republic. kamil.kuca@uhk.cz (K. K).
[6] Biomedical Research Center, University Hospital in Hradec Kralove, Sokolska 581, 50005 Hradec Kralove, Czech Republic;
[7] Laboratory of Computational Chemistry, Department of Chemistry, UFLA, 37200-000, Lavras/MG – Brazil.

* Correspondence: tanos@ime.eb.br (T.C.C.F); kamil.kuca@uhk.cz (K. K).

Supplementay Material

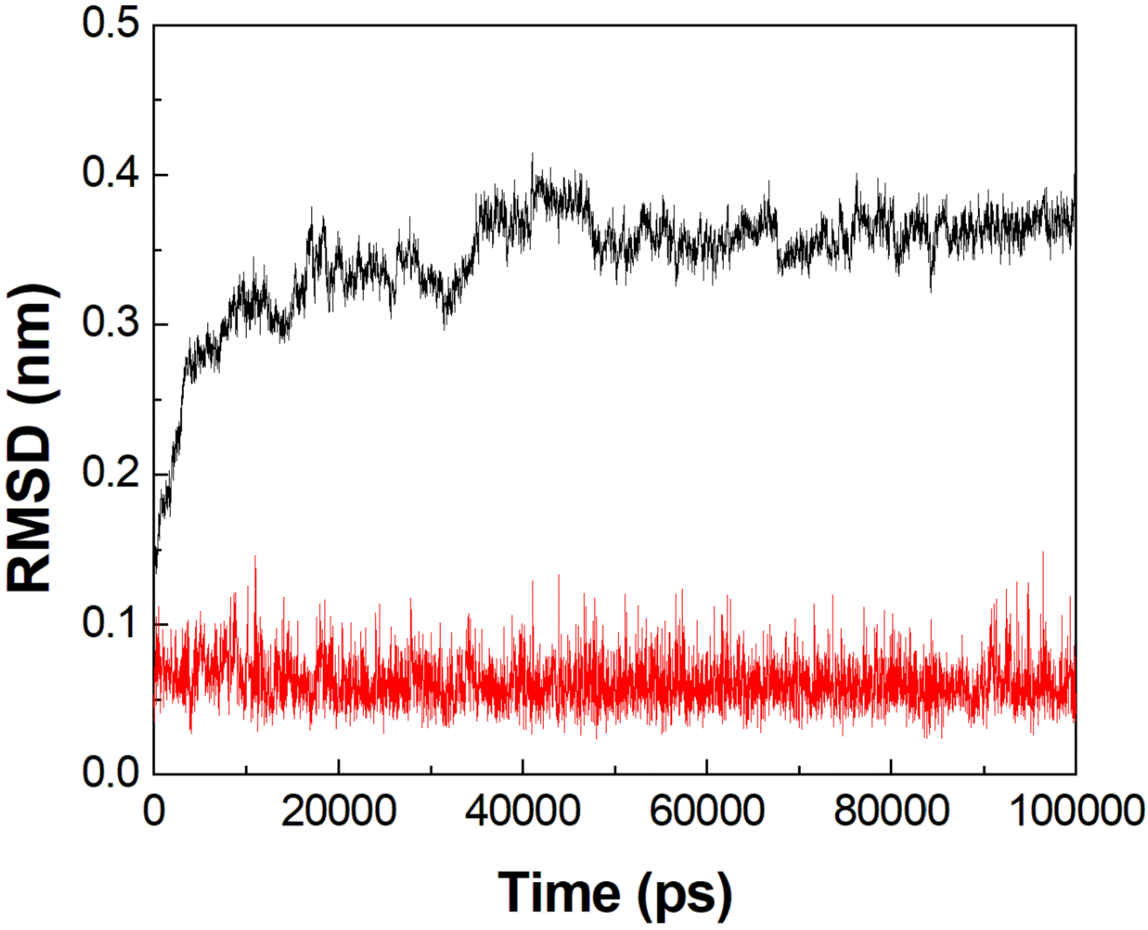

Figure S1 - RMSD of the first MD simulation for the system HssTMPK/Compound 2. Black - Protein; Red - Compound 2.

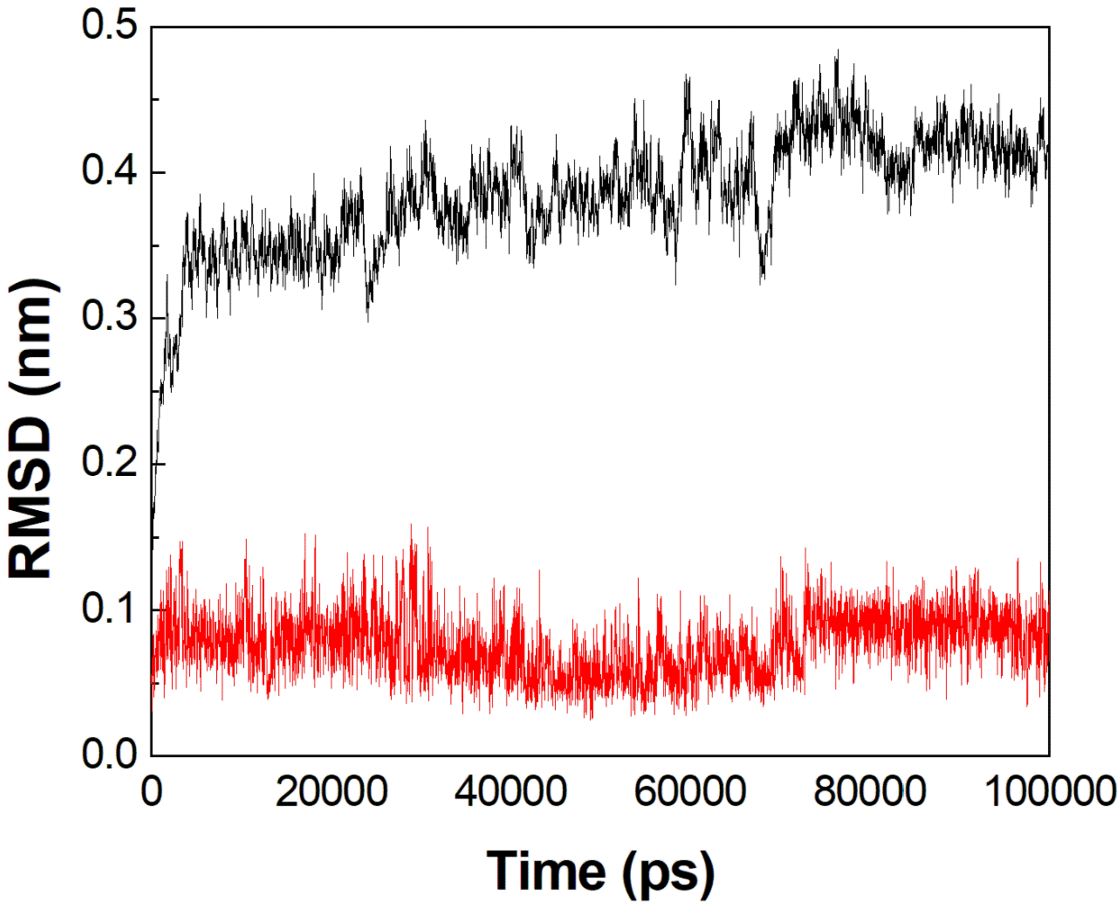

Figure S2 - RMSD of the second MD simulation for the system HssTMPK/Compound 2. Black - Protein; Red - Compound 2.

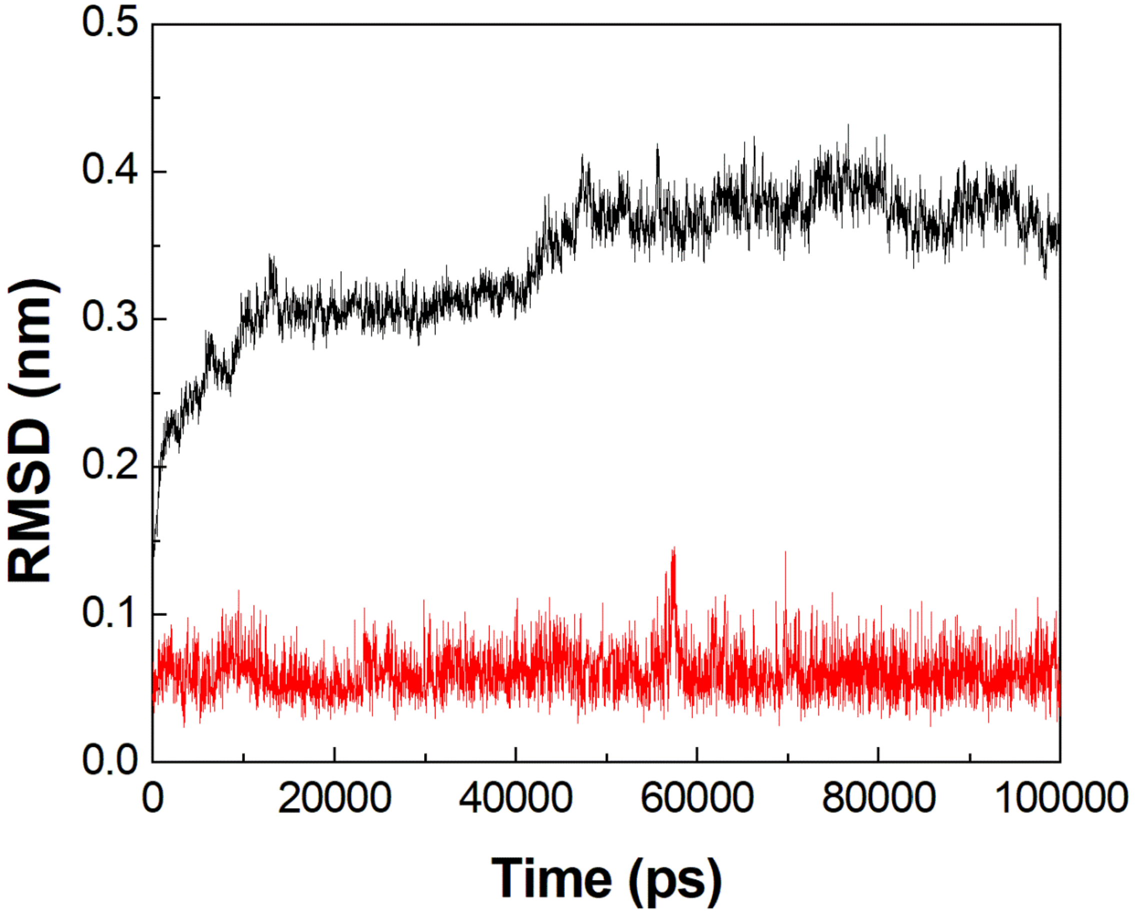

Figure S3 - RMSD of the third MD simulation for the system HssTMPK/Compound 2. Black - Protein; Red - Compound 2.

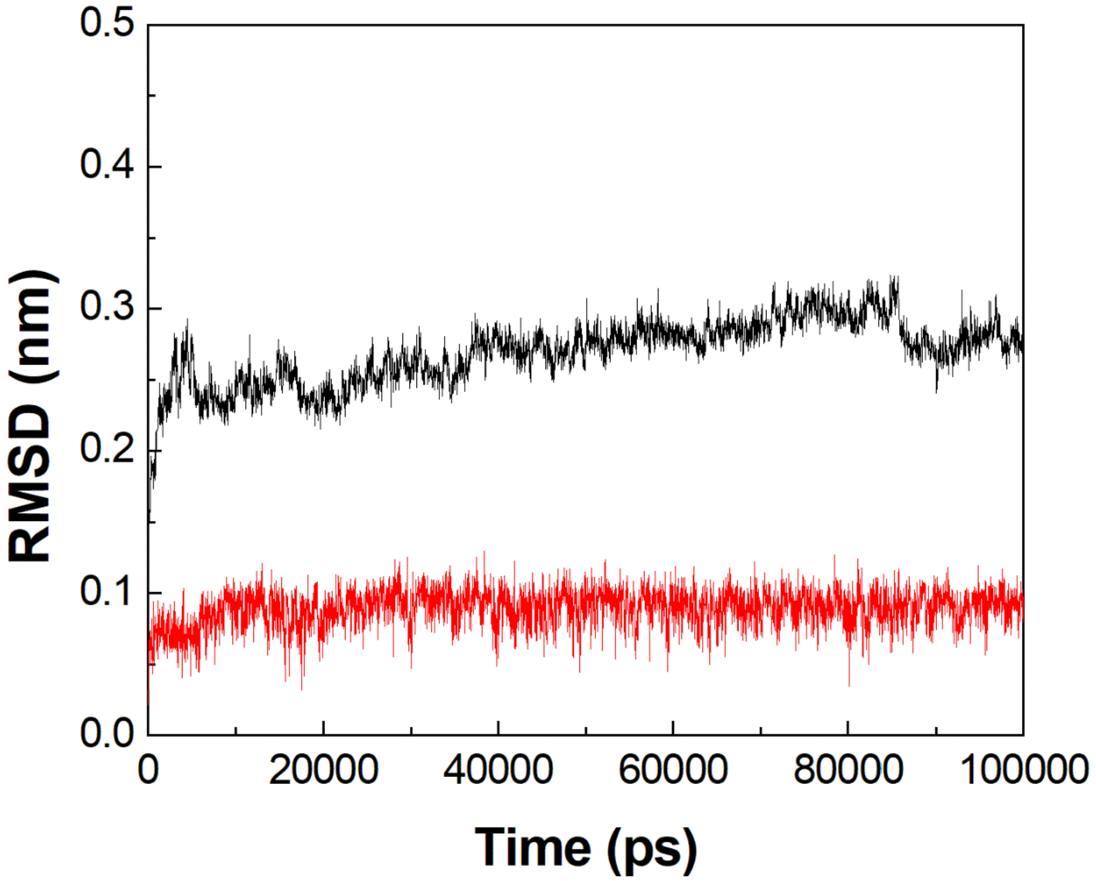

Figure S4 - RMSD of the first MD simulation for the system VarTMPK/Compound 2. Black - Protein; Red - Compound 2.

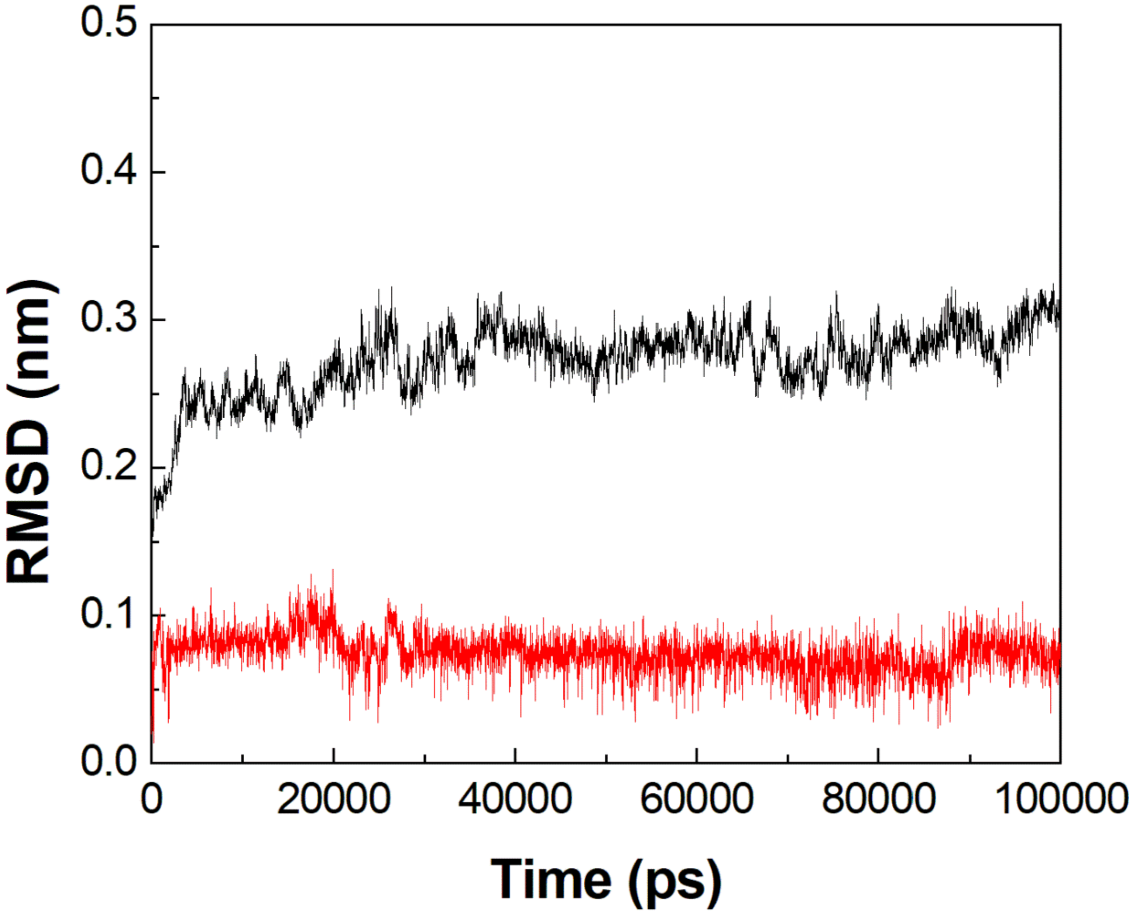

Figure S5 - RMSD of the second MD simulation for the system VarTMPK/Compound 2. Black - Protein; Red - Compound 2.

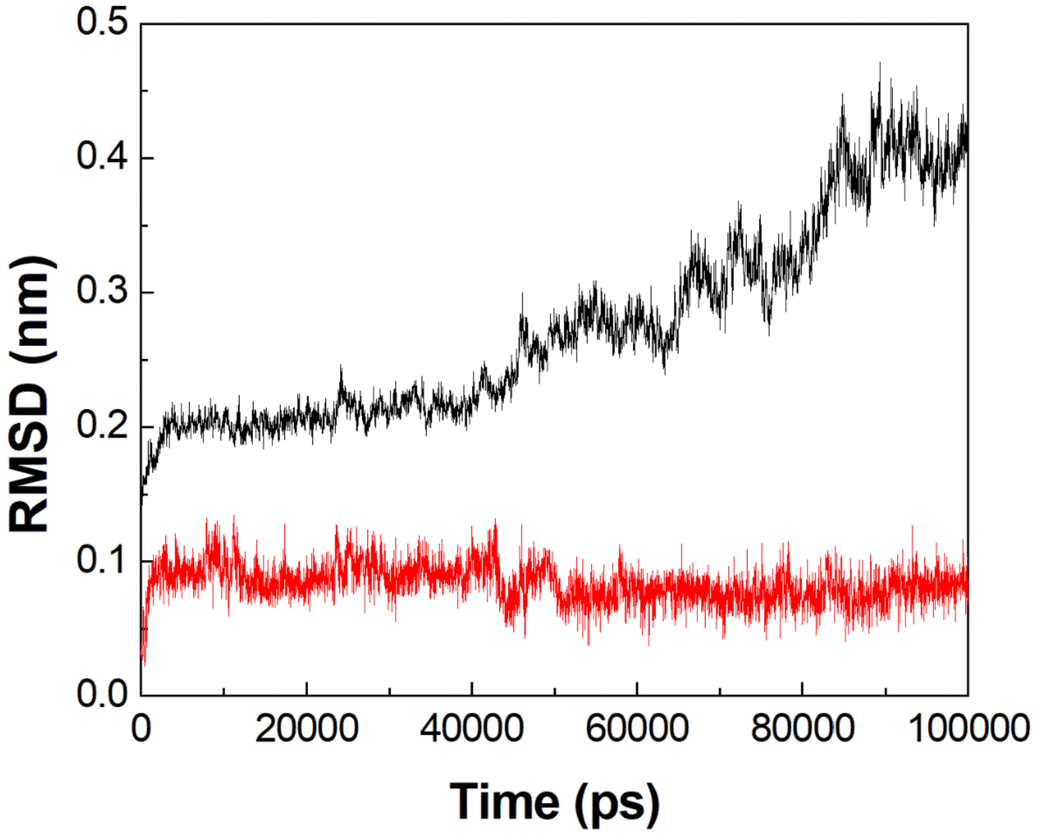

Figure S6 - RMSD of the third MD simulation for the system VarTMPK/Compound 2. Black - Protein; Red - Compound 2.
